# Effect of eight-form Tai Chi combined with olfactory stimulation on working memory in older adults with mild cognitive impairment

**DOI:** 10.3389/fpubh.2025.1724647

**Published:** 2026-01-06

**Authors:** Jialei Huang, Chunhui Zhou, Miao Wang, Hua Yang, Ganfeng Yang

**Affiliations:** School of Physical Education and Sports Science, Soochow University, Suzhou, China

**Keywords:** mild cognitive impairment, older adults, olfactory stimulation, Tai Chi, working memory

## Abstract

**Background:**

Tai Chi integrates multisensory stimuli, including olfactory cues, to enhance cognitive functions such as working memory. This study evaluated the effects of eight-form Tai Chi combined with olfactory stimulation on working memory in older adults with mild cognitive impairment (MCI).

**Methods:**

This study used a 2 (time) × 3 (group) mixed design. Of 267 screened patients with MCI, 93 were deemed eligible and randomly assigned to one of three groups: Tai Chi group (TCG), Tai Chi combined with olfactory stimulation group (TCOG), or control group (CG). The TCG and TCOG participated in three weekly 60 min sessions for 26 weeks. The TCOG was exposed to four fragrances. Primary outcomes were the N-back task and the digit span test (DST). Secondary outcomes included the MoCA, MMSE, CSIT, GDS, SAS, and PSQI.

**Results:**

Post-intervention, both intervention groups outperformed the CG in 0-back accuracy (TCG: +0.118, 95% CI: 0.048–0.188, *p* < 0.001; TCOG: +0.118, 95% CI: 0.048–0.187, *p* < 0.001) and total DST scores (TCG: +1.321, 95% CI: 0.250–2.393, *p* = 0.009; TCOG: +1.535, 95% CI: 0.602–2.467, *p* < 0.001). They also exhibited significant improvements in MoCA (TCG: +3.07, 95% CI: 0.89–5.25, *p* = 0.002; TCOG: +3.42, 95% CI: 1.22–5.62, *p* = 0.001) and CSIT scores (TCG: +3.29, 95% CI: 1.56–5.01, *p* < 0.001; TCOG: +3.86, 95% CI: 2.28–5.44, *p* < 0.001) compared with the CG. Furthermore, the TCOG showed superior gains over the TCG in 1-back accuracy (+0.156, 95% CI: 0.044–0.269, *p* = 0.003), forward digit span (+0.613, 95% CI: 0.156–1.070, *p* = 0.004), and backward digit span (+0.921, 95% CI: 0.117–1.726, *p =* 0.018). It likewise showed significantly greater reductions in GDS (−1.46, 95% CI: −2.78 to −0.15, *p* = 0.024) and PSQI scores (−2.04, 95% CI: −3.89 to −0.19, *p* = 0.008) compared with the other groups, with the TCOG versus TCG in PSQI being significant (−1.47, 95% CI: −2.98 to −0.04, *p* = 0.020).

**Conclusion:**

Tai Chi improved working memory and global cognition in older adults with MCI. Combining Tai Chi with olfactory stimulation yielded additional benefits, demonstrating superior efficacy in alleviating depressive symptoms and enhancing sleep quality.

**Clinical trial registration:**

https://www.chictr.org.cn/index.html, Chinese Clinical Trial Registry (ChiCTR2400083424).

## 1 Introduction

Amid the acceleration of global population aging, the presence of mild cognitive impairment (MCI) has emerged as a significant risk factor affecting the health of older adults (1, 2). Individuals with MCI typically exhibit a marked decline in cognitive abilities across multiple domains, including memory, attention, and executive function. This not only severely impacts their quality of daily life but also imposes increasing demands on family and societal care resources. Consequently, the effective prevention and management of MCI has become a significant public health challenge within the field of geriatric health.

Tai Chi has gained widespread recognition as an effective intervention for improving emotional regulation and enhancing cognitive function. Research indicates that this practice aids in strengthening physical capabilities, alleviating symptoms of chronic conditions, improving balance and gait stability, and reducing emotional issues such as anxiety and depression. In terms of cognitive function, Tai Chi has been shown to promote several cognitive dimensions. Tai Chi may improve overall cognitive function by promoting cerebral blood flow and enhancing neural activity (3). Long-term practice is strongly associated with improvements in memory, attention, executive function, and visuospatial abilities, and these benefits are thought to result from neuroadaptive changes in the function and structure of the relevant brain areas (4, 5). Among them, working memory, as a core component of executive function, is susceptible to deterioration during aging. Randomized controlled trials have proven that Tai Chi significantly improves working memory performance, especially in older adults with MCI (6). Neurological studies have shown that Tai Chi enhances neuroplasticity through slow, coordinated movements, attentional regulation, and breathing, and this improvement may be related to enhanced activation of the prefrontal cortex and increased efficiency of neural information processing, thereby optimizing the encoding and extraction of working memory (7–10). In recent years, multimodal intervention strategies have been gaining attention. Compared with single training, the combination of Tai Chi and sensory stimulation may produce synergistic benefits to enhance emotional and memory functions. In this context, a “synergistic” effect denotes a combined intervention outcome that exceeds the sum of improvements achievable by each component administered separately. This enhanced efficacy, particularly evident in working memory performance, presumably arises from the engagement of complementary physiological and neural pathways through multimodal integration. During exercise, participants receive external information, and the brain improves neural arousal and cognitive engagement through multisensory integration.

Notably, olfactory-related brain regions become structurally and functionally abnormal earlier in the progression from subjective cognition decline to MCI, and they continue to worsen as AD progresses (11). Olfactory recognition deficits are closely related to AD pathology and often precede the onset of significant memory deficits, making them reliable and non-invasive predictors. Studies have demonstrated that deficits in olfactory recognition indicate neurodegenerative changes mediated by the tau protein. These deficits are linked to various pathological outcomes, such as cognitive decline, atrophy of the medial temporal lobe, and amyloid deposition in individuals with MCI (12). Based on the above mechanisms, olfactory stimulation has been proposed as a potential non-pharmacological intervention. A seminal study by Rasch et al. offers a clear experimental paradigm. In their procedure, rose odor was delivered via an olfactometer during the slow-wave sleep of participants. Improvement in visuospatial working memory, as measured by a 2-back task, was interpreted as resulting from the odor-induced reactivation and consolidation of hippocampal memory traces. The olfactometer provided controlled odor stimulation, enabling precise delivery while isolating olfactory input from tactile or auditory interference (13). This result aligns with broader evidence showing that controlled olfactory exposure can activate memory-related brain regions and enhance cognitive function (14). Regular exposure to specific odors, especially during sleep, strengthens hippocampal–prefrontal loop functional connectivity and promotes working memory consolidation (11). Structured olfactory training, defined as the repeated and systematic exposure to a set of distinct odorants over several weeks to months, has also been shown to improve verbal and spatial working memory, suggesting that the intervention can directly modulate the information-processing capacity of key neural networks (15).

Although exercise interventions and sensory stimulation have demonstrated positive effects in improving cognitive function among individuals with MCI, existing research exhibits notable limitations. The majority of current studies focus on single-intervention models, independently examining the effects of either exercise or sensory stimulation, with a lack of systematic exploration into their synergistic effects (16). From a mechanistic perspective, exercise intervention primarily exerts its effects through pathways such as promoting cerebral blood flow, enhancing neuroplasticity, and regulating brain-derived neurotrophic factor (BDNF) levels (17). By contrast, olfactory stimulation emphasizes activating the olfactory bulb–hippocampal pathway, strengthening functional connectivity between olfactory and limbic structures, and modulating neuro-oscillatory activity associated with cognition (18). These two approaches exhibit distinct complementary mechanisms, theoretically capable of producing synergistic gains through multi-system, multi-target integration. However, empirical research on combined exercise and olfactory interventions remains limited, and their synergistic mechanisms, optimal intervention parameters, and suitable populations have yet to be clarified (19). Therefore, multimodal intervention studies are necessary to systematically evaluate whether the combination of eight-form Tai Chi and olfactory stimulation can effectively improve cognitive functions such as working memory in older adults with MCI through multi-pathway synergistic effects. This would provide theoretical and practical references for developing personalized, integrated non-pharmacological intervention programs.

In summary, this study aimed to systematically investigate the effects of combined eight-form Tai Chi and olfactory stimulation on working memory in older adults with MCI through a randomized controlled trial and comprehensively evaluate the feasibility and participant acceptability of this combined intervention. The study proposes the following primary hypotheses: the combined intervention group will demonstrate significantly greater improvements in working memory compared with the Tai Chi-only group and the control group. Furthermore, both active intervention groups are anticipated to outperform the control group in overall cognitive function, and the combined intervention protocol is expected to exhibit favorable feasibility and participant acceptability.

## 2 Materials and methods

### 2.1 Study design

This study was a single-blind, randomized controlled trial employing a 2 (time: pre-test, post-test) × 3 (CG, TCG, and TCOG) two-factor mixed design. Eligible participants were sequentially numbered upon enrolment and randomly assigned in a 1:1:1 ratio via the REDCap electronic data capture system to the CG, TCG, or TCOG. The allocation sequence was generated by an independent statistician and concealed to ensure randomization and integrity. Outcome evaluators and data analysts were blinded to group assignments to control for assessment bias. The primary outcomes consisted of changes in working memory, cognitive function, and sensory function following 26 weeks of intervention.

### 2.2 Participants

Participants were recruited through word-of-mouth promotion and posters within Suzhou communities. Sample size was estimated using G*Power 3.1.9.7. Through repeated measures ANOVA (effect size *f* = 0.25, test power = 0.95, *α* = 0.05), the calculation indicated a minimum sample size of 66 cases. To account for an estimated 30% attrition rate, we set the final sample size to 31 participants per group, totaling 93 participants. The final analyzed sample consisted of 28 participants in the CG, 28 in the TCG, and 29 in the TCOG. This total represents an actual attrition rate of 8.6%, substantially lower than the conservative 30% estimate used in our initial planning. Importantly, the final sample of 85 participants exceeds the minimum requirement of 66 determined by the power calculation, ensuring maintained statistical power for our primary analyses.

The inclusion criteria were as follows: (1) aged between 60 and 70 years; (2) clinically diagnosed with MCI while maintaining substantial independence in daily living activities; (3) no recent participation in regular physical exercise or similar cognitive intervention programs; (4) absence of harmful lifestyle habits, such as heavy alcohol consumption or smoking, with generally good health; (5) no severe impairment in external sensory perception; and (6) signed informed consent and willingness to participate in the study.

The exclusion criteria included (1) severe cardiovascular disease or contraindications to exercise; (2) diagnosis of severe cognitive impairment, psychiatric disorders, or other neurodegenerative diseases; (3) severe sensory impairments or communication difficulties affecting cognitive assessment; (4) current treatment for cognitive impairment or depression, or use of medications affecting cognitive function; and (5) cognitive decline attributable to intracranial lesions or other neurological conditions (e.g., Parkinson’s disease and stroke).

### 2.3 Randomization and masking

The REDCap system performed randomization to ensure a balanced proportion across the three groups (CG, TCG, and TCOG). The automated allocation module of the system maintained concealment, disclosing group assignment only after completion of baseline assessments and formal enrollment. Given the intervention, blinding of participants and instructors was not feasible. However, outcome assessors and data analysts remained blinded throughout the study. To preserve assessor blinding, evaluations were conducted in dedicated assessment areas physically separated from intervention sites, and participants received explicit instructions regarding non-disclosure of their group assignment. Furthermore, intervention sessions were temporally segregated to minimize intergroup contact and prevent potential contamination. Data analysis adhered to intention-to-treat principles. Researchers performing statistical analyses remained blinded to group allocation until all primary outcome analyses were completed.

### 2.4 Interventions

**Figure 1:**
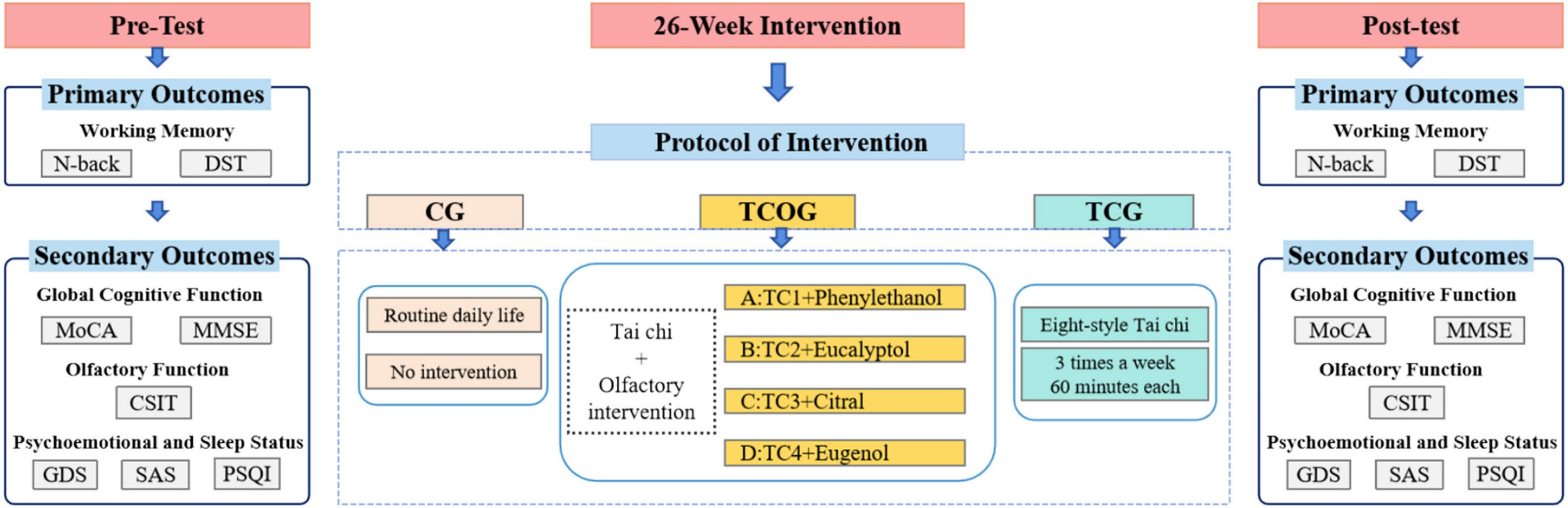
Flowchart of the experimental procedure and assessment timeline.

This study established three intervention groups, and the total intervention duration was 26 weeks. The assessment timeline is outlined in Figure 1. Working memory assessments, along with all other outcome measures, were conducted at two time points: at baseline (pre-test, Week 0) before the initiation of any intervention, and immediately following the completion of the 26-week intervention period (post-test, Week 26). To minimize potential expectancy effects, we gave participants standardized neutral instructions stating that the study aimed to investigate the potential benefits of a combined mind–body and sensory intervention for cognitive health. No specific therapeutic hypotheses were suggested to avoid influencing subjective reporting.

#### 2.4.1 Control group

Participants in this group did not receive any structured physical or cognitive intervention. However, they received equivalent contact time through monthly health education sessions covering general topics unrelated to cognitive training or physical exercise to control for non-specific effects, such as staff attention and social interaction. These sessions were matched in duration to the total contact time of the intervention groups.

#### 2.4.2 Tai Chi group

Participants in this group practiced the eight-form Tai Chi high-posture routine, following recommendations from the Chinese Expert Consensus on Rehabilitation Management of Alzheimer’s Disease (2019). The intervention incorporated four standardized movement sequences to maintain engagement while ensuring consistent technical content. All sequences comprised the same eight core forms: Reverse Reeling Forearm, Brush Knee and Twist Step, Part the Wild Horse’s Mane, Wave Hands Like Clouds, Golden Rooster Stands on One Leg, Kick with Heel, Grasp Sparrow’s Tail, and Cross Hands. These forms were organized into the following sequences:

Sequence 1: Reverse Reeling Forearm → Brush Knee and Twist Step → Part the Wild Horse’s Mane → Wave Hands Like Clouds → Golden Rooster Stands on One Leg → Kick with Heel → Grasp Sparrow’s Tail → Cross Hands.

Sequence 2: Reverse Reeling Forearm → Part the Wild Horse’s Mane → Wave Hands Like Clouds → Golden Rooster Stands on One Leg → Kick with Heel → Grasp Sparrow’s Tail → Brush Knee and Twist Step → Cross Hands.

Sequence 3: Reverse Reeling Forearm → Wave Hands Like Clouds → Golden Rooster Stands on One Leg → Kick with Heel → Grasp Sparrow’s Tail → Brush Knee and Twist Step → Part the Wild Horse’s Mane → Cross Hands.

Sequence 4: Reverse Reeling Forearm → Golden Rooster Stands on One Leg → Kick with Heel → Grasp Sparrow’s Tail → Brush Knee and Twist Step → Part the Wild Horse’s Mane → Wave Hands Like Clouds → Cross Hands.

Sessions consisted of 60 min per session, three times a week, over a period of 26 weeks. Training included warm-up exercises, Tai Chi form practice, and a cool-down relaxation session. All sessions were led by qualified Tai Chi instructors and conducted in standardized venues suitable for older adults. Heart rate and blood pressure were monitored throughout the intervention to ensure safety.

#### 2.4.3 Tai Chi combined with olfactory stimulation group

This group maintained identical Tai Chi training content, duration, frequency, and cycle to the TCG but incorporated olfactory stimulation during practice. Four odor molecules—phenethyl alcohol (rose scent), eucalyptol (eucalyptus scent), citral (lemon scent), and eugenol (clove scent)—were selected based on three criteria: (1) their established use in the validated olfactory training protocol by Hummel et al., ensuring consistency with prior research; (2) their representation of distinct perceptual categories (floral, herbal, citrus, and spicy) to provide broad olfactory stimulation; and (3) their well-characterized safety profiles and widespread use in olfactory research, minimizing adverse effects (20). Odorants were selected based on a pre-study sensory evaluation conducted with an independent panel of healthy volunteers (*n* = 20). Participants rated perceived familiarity, pleasantness, and intensity using Visual Analogue Scales. Only those odorants that consistently demonstrated high familiarity, moderate pleasantness, and medium intensity were adopted, thereby standardizing perceptual characteristics across the study cohort.

To ensure precise and consistent odor delivery, we dissolved pharmaceutical-grade odorants in odorless dipropylene glycol at a standardized 1:10 dilution ratio. Fresh solutions were prepared weekly, stored in amber glass bottles, and maintained at 4 °C to preserve chemical stability. Electric aroma diffusers (AromaMaster-400) were calibrated monthly to ensure consistent output. Ambient odor concentration was maintained within a target range of 0.5–1.0 ppm, verified through periodic measurements using a calibrated portable photoionization detector.

During training, the aroma diffusers released the scents in a pre-set sequence, with each scent delivered for 5 min, followed by a 2 min interval. Overall concentration level was maintained at a perceptible yet non-disruptive threshold. Participants synchronized olfactory inhalation with their Tai Chi practice. To reinforce the odor–exercise context association, we set the intervention across four locations: Location A: eight-form Tai Chi sequence 1 + phenethyl alcohol, Location B: eight-form Tai Chi sequence 2 + eucalyptol, Location C: eight-form Tai Chi sequence 3 + citral, and Location D: eight-form Tai Chi sequence 4 + eugenol. To control for potential confounding effects of familiar and preferred odors, we exposed all participants in the TCOG to all four odors through a standardized rotation schedule. The Tai Chi sequences utilized identical foundational movements but differed in their performance order to maintain cognitive engagement while ensuring consistent physical demands across sessions. Throughout the intervention, operations were supervised by researchers who were uniformly trained and experienced in their field. Portable physiological monitors continuously recorded the heart rates and blood pressures of the participants to ensure the safety and consistency of the intervention.

Adherence and intervention fidelity were rigorously monitored throughout the 26-week study. Participants in the active intervention group completed 85.2% of scheduled sessions. To ensure protocol compliance, all Tai Chi instructors had second-level wushu athlete certification with Tai Chi specialization and completed standardized training in the eight-form routine. Instructional consistency was maintained through biweekly supervision sessions and independent review of randomly selected video recordings. For olfactory administration, aromatherapists prepared odor solutions at standardized concentrations using pharmaceutical-grade ingredients. Identical diffusers were used for automated odor delivery, with proper implementation documented at each session.

### 2.5 Outcome measures

#### 2.5.1 Primary outcomes

##### 2.5.1.1 Assessment of memory function

Working memory capacity was evaluated using the N-back task paradigm. The experimental program was developed using E-Prime 3.0, employing a block design with three conditions categorized by memory load level: 0-back, 1-back, and 2-back. Each condition comprised five repeated blocks. Before the formal experiment, participants completed a practice session to familiarize themselves with task requirements. During the experiment, subjects were instructed to respond as rapidly and accurately as possible. The entire task duration was approximately 15 min. Behavioral data were collected and organized using E-Data, with mean reaction times and accuracy rates calculated for each participant across load conditions. Final data were exported to Excel for subsequent statistical analysis.

The digit span test (DST), a key component of the Wechsler Adult Intelligence Scale, was employed to assess working memory and attentional functions. It comprised two sections: forward and backward. In the forward task, the examiner read aloud a progressively increasing sequence of digits at a rate of one digit per second (e.g., 4-7-2-1). The subject must repeat the sequence in the same order. The test involved eight items, with one point awarded for each correct response. This section primarily assessed short-term memory and auditory attention. In the backward task, the subject must repeat the heard sequence in reverse order (e.g., repeating “4-7-2-1” backwards as “1-2-7-4”). This also comprised eight items, with one point awarded for each correct response. This task placed increased demands on working memory, cognitive flexibility, and central executive function. The final total score was derived by summing the forward and backward scores (maximum of 16 points).

#### 2.5.2 Secondary outcomes

##### 2.5.2.1 Global cognitive function

Montreal Cognitive Assessment (MoCA) and Mini-Mental State Examination (MMSE) were used to evaluate overall cognitive function (21, 22). The MoCA assesses eight cognitive domains: visuospatial and executive function, naming, memory, attention, language, abstract reasoning, delayed recall, and orientation. The MMSE primarily evaluates orientation, immediate memory, attention and calculation, recall, naming, repetition, reading, three-step commands, expression, and drawing. The MoCA exhibits high sensitivity in detecting MCI, whereas the MMSE demonstrates high specificity in screening moderate to severe cognitive impairment. The MoCA accounts for educational influence by adding one point for each additional year of schooling (maximum score of 30). The MMSE employs distinct cutoff scores based on educational attainment: 17 points for individuals who are illiterate, 20 points for primary school graduates, and 24 points for those with secondary education or higher. Combining these instruments enables increased adaptation to diverse educational backgrounds, offering accurate and comprehensive cognitive assessments while minimizing the possibility of overlooking potential cognitive deficits through single-test evaluation.

##### 2.5.2.2 Olfactory function

The Chinese Smell Identification Test (CSIT), developed by the Institute of Psychology at the Chinese Academy of Sciences, was employed. This test takes approximately 15 min to complete (23). All participants underwent testing in a quiet, well-ventilated environment. They were required to fast for 30 min before testing but were permitted to drink plain water. During the test, the lid of the olfactory testing device (Model: XLX-CSIT-STK-16, Ming Shi Technology (Suzhou) Co., Ltd.) was opened, and the device was positioned approximately 1 cm from the participant’s nasal tip. The device was then shaken 5–7 times, after which participants were required to identify the odor from four options. In cases of uncertainty, participants were obliged to make a choice. A 30 s interval was observed between each test to prevent olfactory fatigue. The test comprised 16 familiar odors readily recognizable to Chinese individuals. Each correctly identified odor yielded 1 point, with a maximum achievable score of 16 points.

##### 2.5.2.3 Psychoemotional and sleep status

The Geriatric Depression Scale (GDS) was developed by Yesavage et al. (24) to screen for depressive symptoms in older adults. This study employed the 30-item version, with participants responding “yes” or “no” based on their feelings over the past week. A total score of 0–10 indicates normal functioning, 11–20 denotes mild depression, and 21–30 signifies moderate to severe depression (24). The Self-Rating Anxiety Scale (SAS), developed by Zung (34), comprises 20 items reflecting physical and psychological anxiety symptoms. It employs a four-point rating scale. The sum of individual item scores is multiplied by 1.25 to yield a standardized score. A score below 50 indicates normal anxiety levels, 50–60 indicates mild anxiety, 61–70 denotes moderate anxiety, and scores above 70 signify severe anxiety (25). The Pittsburgh Sleep Quality Index (PSQI), developed by Buysse et al., (35) comprises 19 self-report items assessing sleep quality across seven dimensions: subjective sleep quality, sleep onset latency, sleep duration, sleep efficiency, sleep disturbances, hypnotic medication use, and daytime dysfunction. Each dimension is scored from 0 to 3, yielding a total score ranging from 0 to 21. Higher scores indicate poorer sleep quality. A score of 0–5 indicates good sleep quality, 6–10 denotes moderate sleep disturbance, and ≥11 signifies severe sleep disturbance (26).

### 2.6 Ethical review and informed consent

This research protocol was approved by the Ethics Committee of Soochow University (Approval no. SUDA20221106H02), with all procedures adhering to the latest revision of the Declaration of Helsinki, and registered with the Chinese Clinical Trial Registry (Registration no. ChiCTR2400083424). Before commencement, researchers provided participants with detailed information regarding the content and objectives of the study, ensuring that all data would be used solely for academic research and publication purposes while maintaining the strict confidentiality of personal information. Participants were explicitly informed of their right to withdraw from the study at any time without any penalty. All participants signed informed consent forms.

### 2.7 Statistical analysis

Data analysis for this study was conducted using IBM SPSS Statistics 26.0. For continuous variables that met the assumptions of normal distribution and homogeneity of variance, results are presented as mean ± standard deviation (mean ± SD), with intergroup comparisons performed using one-way ANOVA. Variables not meeting normality or homogeneity of variance were expressed as median (interquartile range) [M(IQR)], with intergroup comparisons conducted using the Kruskal–Wallis *H* test. Categorical variables were described by frequency (percentage) [*n* (%)], with intergroup comparisons performed using chi-square tests. To evaluate the efficacy of the intervention, this study employed generalized estimating equation (GEE) to analyze repeated measures data, examining the effects of time, group, and their interaction. The GEE model was applied with an exchangeable working correlation structure. This method incorporates all available data under the assumption that missing values are missing completely at random (MCAR), an assumption supported by Little’s MCAR test (*χ*^2^ = 5.32, *p* = 0.256). Analyses adhered to the intention-to-treat principle by including all randomized participants. In cases where significant interaction or main effects were identified, post-hoc pairwise comparisons were performed using Bonferroni adjustment to maintain the family-wise error rate. Sensitivity analyses based on multiple imputation for missing data also produced results consistent with those from the primary GEE analysis, underscoring the robustness of the findings. Statistical significance was set at *p* < 0.05.

## 3 Results

### 3.1 Baseline characteristics of participants

**Figure 2:**
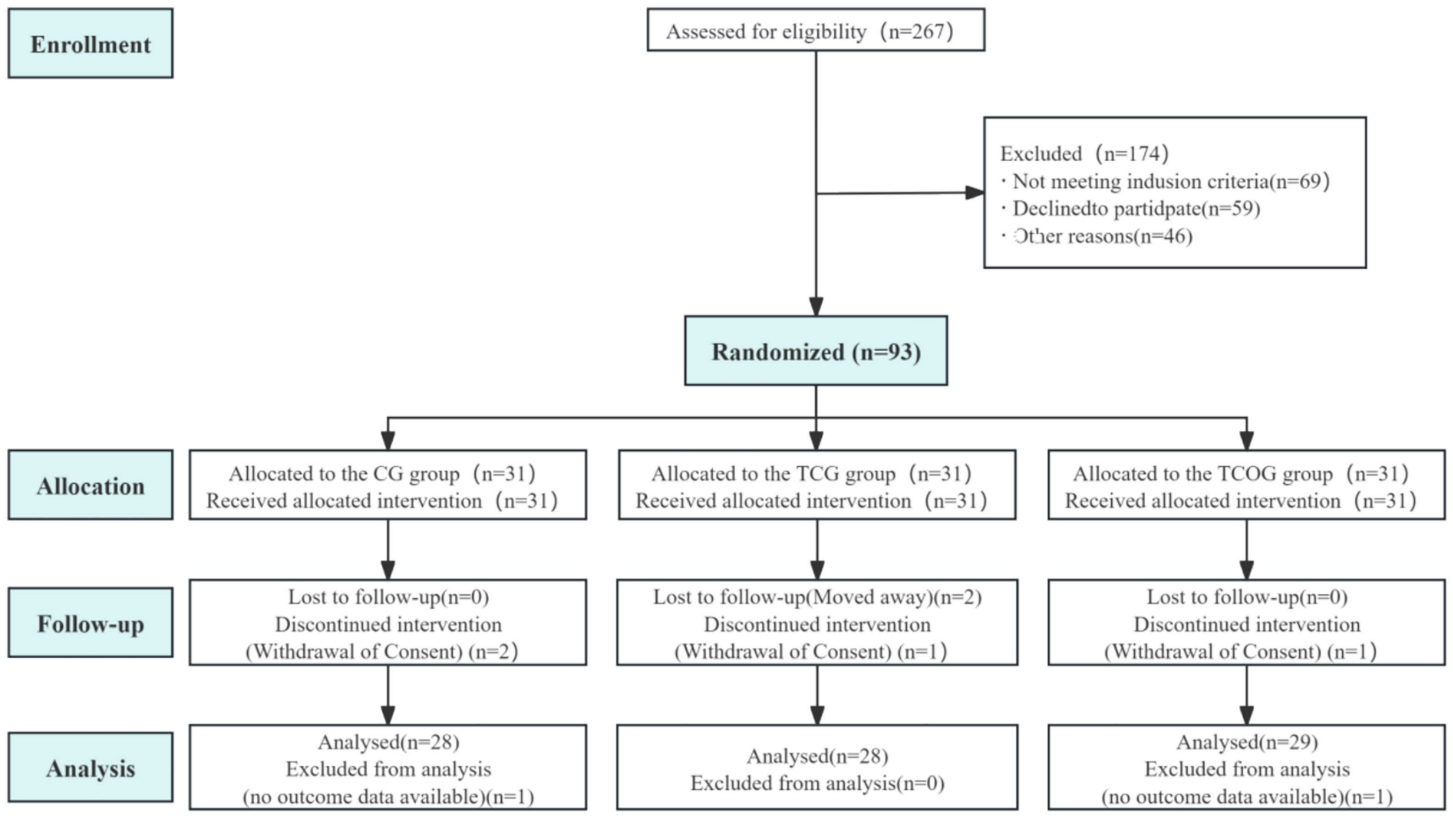
Schematic presentation of participant screening, randomization, and intervention.

Of the 267 participants initially screened, 93 met the inclusion criteria and voluntarily participated in the study. The study flow diagram is presented in Figure 2. Participants were randomly assigned to three groups: CG (*n* = 28), TCG (*n* = 28), and TCOG (*n* = 29). During the 26-week intervention period, eight participants dropped out: 3 from the CG (2 lost to follow-up, 1 missing data), 3 from the TCG (2 discontinued intervention, 1 self-withdrew), and 2 from the TCOG (1 discontinued intervention, 1 missing data). Overall attendance was approximately 85%. Analysis of baseline data for subjects in all three groups before intervention revealed no significant differences (*p* > 0.05) across groups in terms of gender, age, years of education, N-back accuracy and reaction time, DST, MoCA, MMSE, olfactory function, depression, anxiety, or sleep (Table 1).

**Table 1:** Baseline characteristics of participants.

Variables	CG (*N* = 28)	TCG (*N* = 28)	TCOG (*N* = 29)	*X*^2^/*H*/*F*	*P*-value
Gender (Male/Female)	13(46.4)/15(53.6)	9(32.1)/19(67.9)	9(31.0)/20(69.0)	1.795^b^	0.408
Age (years)	67.50 (7.50)	66.50 (5.75)	67.00 (4.00)	1.341	0.511
Years of education (years)	9.00 (2.75)	10.50 (6.00)	9.00 (4.50)	4.157	0.125
0-back ACC	0.95 (0.05)	0.97 (0.05)	0.95 (0.05)	2.249	0.325
0-back RT (ms)	571.00 (245.00)	676.00 (185.50)	690.00 (283.00)	2.391	0.303
1-back ACC	0.86 (0.47)	0.88 (0.43)	0.78 (0.19)	0.666	0.717
1-back RT (ms)	908.50 (368.00)	787.00 (297.00)	889.00 (321.50)	1.016	0.602
2-back ACC	0.57 ± 0.17	0.65 ± 0.21	0.61 ± 0.18	1.174^a^	0.314
2-back RT (ms)	1109.57 ± 332.16	1173.43 ± 350.60	1198.41 ± 269.67	0.585^a^	0.559
Forward (scores)	7.00 (2.00)	7.00 (1.00)	7.00 (1.00)	0.345	0.842
Backward (scores)	3.50 (2.00)	4.00 (1.00)	4.00 (1.00)	0.058	0.971
DST (scores)	10.00 (3.75)	11.00 (2.75)	10.00 (2.00)	0.024	0.988
MoCA (scores)	20.00 (5.50)	21.50 (3.00)	22.00 (5.00)	3.700	0.157
MMSE (scores)	24.50 (2.00)	25.00 (1.75)	25.00 (1.50)	1.307	0.520
CSIT (scores)	12.07 ± 1.90	12.46 ± 2.41	12.72 ± 1.91	0.706^a^	0.497
GDS (scores)	4.61 ± 2.33	4.39 ± 1.69	4.83 ± 2.05	0.323^a^	0.725
SAS (scores)	31.00 (5.00)	32.00 (9.00)	32.00 (5.00)	1.337	0.513
PSQI (scores)	5.00 (4.00)	6.00 (3.75)	5.00 (4.50)	0.288	0.866

^a^One-way analysis of variance.

^b^Chi-square test.

CG, control group; TCG, Tai Chi group; TCOG, Tai Chi combined with olfactory intervention group; ACC, accuracy; RT, reaction time; DST, digit span test; MoCA, Montreal Cognitive Assessment; MMSE, Mini-Mental State Examination; CSIT, Chinese Smell Identification Test; GDS, Geriatric Depression Scale; SAS, Self-Rating Anxiety Scale; PSQI (scores), Pittsburgh Sleep Quality Index.

### 3.2 Primary outcome measure results

Primary outcomes were assessed at baseline (T0) and after the 26-week intervention (T1). The 0-back task accuracy revealed a significant interaction between group and time (Wald *χ*^2^ = 15.859, *p* < 0.001). *Post hoc* analyses revealed that both intervention groups demonstrated significantly greater improvements in post-test accuracy from T0 to T1 compared with the CG (TCOG vs. CG: mean difference = 0.118, 95% CI: 0.048–0.188, *p* < 0.001; TCG vs. CG: mean difference = 0.118, 95% CI: 0.048–0.187, *p* < 0.001). The difference between the two intervention groups was not statistically significant (mean difference = 0.000, 95% CI: −0.019 to 0.019, *p* = 1.000) (Tables 2, 3).

**Table 2:** Summary of generalized estimated equation analysis.

Measurement	Time	Group			Interaction effect		Group effect		Time effect	
		CG	TCG	TCOG	Wald *X*^2^	*P*	Wald *X*^2^	*P*	Wald *X*^2^	*P*
Primary outcomes										
0-back ACC	T0	0.94 ± 0.03	0.95 ± 0.04	0.94 ± 0.03	15.859	<0.001^***^	16.065	<0.001^***^	2.871	0.090
	T1	0.85 ± 0.15	0.97 ± 0.03	0.97 ± 0.03						
0-back RT (ms)	T0	698.64 ± 281.63	722.39 ± 214.10	692.41 ± 135.53	7.083	0.029^*^	3.933	0.140	0.112	0.737
	T1	832.07 ± 392.47	680.71 ± 197.67	627.76 ± 125.49						
1-back ACC	T0	0.74 ± 0.24	0.75 ± 0.21	0.76 ± 0.13	6.912	0.032^*^	4.744	0.093	8.384	0.004^**^
	T1	0.72 ± 0.25	0.86 ± 0.18	0.88 ± 0.06						
1-back RT (ms)	T0	940.93 ± 295.20	929.57 ± 361.06	909.41 ± 180.05	3.175	0.204	2.111	0.348	1.041	0.307
	T1	989.82 ± 371.58	828.43 ± 304.86	867.66 ± 106.29						
2-back ACC	T0	0.57 ± 0.17	0.65 ± 0.21	0.61 ± 0.18	4.339	0.114	16.144	<0.001^***^	8.357	0.114
	T1	0.57 ± 0.18	0.77 ± 0.15	0.70 ± 0.14						
2-back RT (ms)	T0	1109.57 ± 332.16	1173.43 ± 350.60	1198.41 ± 269.67	8.749	0.013^*^	0.071	0.965	15.750	<0.001^***^
	T1	1117.32 ± 349.61	1010.54 ± 296.18	1006.52 ± 194.63						
Forward (scores)	T0	6.43 ± 0.92	6.50 ± 0.84	6.66 ± 0.61	8.844	0.012^*^	5.398	0.067	0.380	0.538
	T1	6.21 ± 0.96	6.68 ± 0.61	6.83 ± 0.38						
Backward (scores)	T0	3.61 ± 1.20	3.61 ± 0.74	3.48 ± 1.21	10.368	0.006^**^	3.138	0.208	4.324	0.038^*^
	T1	3.29 ± 1.51	4.14 ± 1.43	4.21 ± 1.01						
DST (scores)	T0	10.04 ± 1.86	10.11 ± 1.40	10.14 ± 1.43	17.258	<0.001^***^	5.276	0.071	3.945	0.047^*^
	T1	9.50 ± 1.71	10.82 ± 1.70	11.03 ± 1.24						
Secondary outcomes										
MoCA (scores)	T0	20.21 ± 2.96	21.61 ± 1.93	21.34 ± 2.79	52.241	<0.001^***^	48.462	<0.001^***^	13.363	<0.001^***^
	T1	17.79 ± 3.75	24.79 ± 3.70	25.17 ± 3.82						
MMSE (scores)	T0	24.14 ± 1.51	24.68 ± 0.98	24.55 ± 1.09	8.958	0.011^*^	17.292	<0.001^***^	16.548	<0.001^***^
	T1	23.75 ± 4.68	27.17 ± 1.65	27.17 ± 1.65						
CSIT (scores)	T0	12.07 ± 1.90	12.46 ± 2.41	12.72 ± 1.91	17.866	<0.001^***^	27.077	<0.001^***^	0.010	0.919
	T1	10.07 ± 3.24	13.36 ± 2.15	13.93 ± 1.49						
GDS (scores)	T0	4.61 ± 2.33	4.39 ± 1.69	4.83 ± 2.05	21.505	<0.001^***^	2.789	0.248	1.527	0.217
	T1	5.36 ± 2.34	3.68 ± 3.42	3.90 ± 1.84						
SAS (scores)	T0	30.96 ± 3.29	32.68 ± 5.33	31.08 ± 3.76	2.595	0.273	4.747	0.093	3.613	0.057
	T1	30.96 ± 4.89	31.82 ± 4.99	29.31 ± 2.89						
PSQI (scores)	T0	6.21 ± 3.35	6.14 ± 2.22	6.17 ± 2.95	8.095	0.017^*^	2.907	0.234	9.384	0.002^**^
	T1	6.21 ± 3.70	5.64 ± 2.82	4.17 ± 1.95						

CG, control group; TCG, Tai Chi group; TCOG, Tai Chi combined with olfactory intervention group; ACC, accuracy; RT, reaction time; DST, digit span test; MoCA, Montreal Cognitive Assessment; MMSE, Mini-Mental State Examination; CSIT, Chinese Smell Identification Test; GDS, Geriatric Depression Scale; SAS, Self-Rating Anxiety Scale; PSQI, Pittsburgh Sleep Quality Index; *P < 0.05, **p < 0.01, ***P < 0.001.

**Table 3:** Bonferroni corrected pairwise comparisons between groups.

Measurement	Comparison	Mean adjusted change (95% CI)	*p-*value
Primary outcomes			
0-back ACC	TCOG vs. CG	0.118 (0.048 to 0.188)	<0.001^***^
	TCG vs. CG	0.118 (0.048 to 0.187)	<0.001^***^
	TCOG vs. TCG	0.000 (−0.019 to 0.019)	1
0-back RT (ms)	TCOG vs. CG	−204.313 (−387.088 to −21.538)	0.022^*^
	TCG vs. CG	−151.357 (−346.586, 43.872)	0.19
	TCOG vs. TCG	52.956 (−50.568 to 156.479)	0.662
1-back ACC	TCOG vs. CG	0.156 (0.044 to 0.269)	0.003**
	TCG vs. CG	0.134 (−0.002 to 0.271)	0.056
	TCOG vs. TCG	0.022 (−0.063 to 0.107)	1
2-back RT (ms)	TCOG vs. CG	−110.80 (−287.87 to 66.26)	0.402
	TCG vs. CG	−106.79 (−310.35 to 96.78)	0.628
	TCOG vs. TCG	−4.02 (−160.68 to 152.64)	1
Forward	TCOG vs. CG	0.613 (0.156 to 1.070)	0.004^*^
	TCG vs. CG	0.464 (−0.040 to 0.969)	0.083
	TCOG vs. TCG	0.149 (−0.171 to 0.469)	0.793
Backward	TCOG vs. CG	0.921 (0.117 to 1.726)	0.018^*^
	TCG vs. CG	0.857 (−0.068 to 1.783)	0.08
	TCOG vs. TCG	0.064 (−0.711 to 0.839)	1
DST	TCOG vs. CG	1.535 (0.602 to 2.467)	<0.001^***^
	TCG vs. CG	1.321 (0.250 to 2.393)	0.009^**^
	TCOG vs. TCG	0.213 (−0.716 to 1.142)	1
Secondary outcomes			
MoCA	TCOG vs. CG	7.387 (5.031 to 9.743)	<0.001^***^
	TCG vs. CG	7.000 (4.663 to 9.338)	<0.001^***^
	TCOG vs. TCG	0.387 (−1.954 to 2.727)	1
MMSE	TCOG vs. CG	3.422 (1.222 to 5.623)	0.001^**^
	TCG vs. CG	3.071 (0.889 to 5.254)	0.002^**^
	TCOG vs. TCG	−0.351 (−1.330 to 0.628)	1
CSIT	TCOG vs. CG	3.860 (2.280 to 5.440)	<0.001^***^
	TCG vs. CG	3.286 (1.558 to 5.013)	<0.001^***^
	TCOG vs. TCG	0.574 (−0.580 to 1.728)	0.701
GDS	TCOG vs. CG	−1.461 (−2.776 to −0.145)	0.024^*^
	TCG vs. CG	−1.679 (−3.521 to 0.164)	0.088
	TCOG vs. TCG	0.218 (−1.501 to 1.937)	1
PSQI	TCOG vs. CG	−2.042 (−3.891 to −0.193)	0.008^**^
	TCG vs. CG	−0.571 (−2.636 to 1.493)	0.508
	TCOG vs. TCG	−1.470 (−2.984 to 0.043)	0.020^*^

CG, control group; TCG, Tai Chi group; TCOG, Tai Chi combined with olfactory intervention group; ACC, accuracy; RT, reaction time; DST, digit span test; MoCA, Montreal Cognitive Assessment; MMSE, Mini-Mental State Examination; CSIT, Chinese Smell Identification Test; GDS, Geriatric Depression Scale; SAS, Self-Rating Anxiety Scale; PSQI, Pittsburgh Sleep Quality Index; *P < 0.05, **P < 0.01, ***P < 0.00.

Results for the 0-back task reaction time revealed a significant interaction between reaction time group and time (Wald *χ*^2^ = 7.083, *p* = 0.029). *Post hoc* analysis revealed that the TCOG exhibited significantly greater reduction in reaction time from T0 to T1 compared with the CG (mean difference = −204.31, 95% CI: −387.09 to −21.54, *p* = 0.022). The difference between the TCG and CG did not reach statistical significance (mean difference = −151.36 ms, 95% CI: −346.59 to 43.87, *p* = 0.190).

Significant group × time interaction effects (Wald *χ*^2^ = 6.912, *p* = 0.032) and significant main time effects (Wald *χ*^2^ = 8.384, *p* = 0.004) were observed in 1-back task accuracy. Post-hoc comparisons revealed that the TCOG demonstrated significantly greater improvement in accuracy from T0 to T1 compared with the CG (mean difference = 0.156, 95% CI: 0.044–0.269, *p* = 0.003). The difference between the TCG and CG was borderline and did not reach statistical significance (mean difference = 0.134, 95% CI: −0.002 to 0.271, *p* = 0.056).

The interaction between group and time for the total DST score was statistically significant (Wald *χ*^2^ = 17.258, *p* < 0.001). *Post hoc* analysis indicated that both intervention groups demonstrated significantly greater score improvement from T0 to T1 than the CG (TCOG vs. CG: mean difference = 1.535, 95% CI: 0.602–2.467, *p* < 0.001; TCG vs. CG: mean difference = 1.321, 95% CI: 0.250–2.393, *p* = 0.009).

The forward group exhibited a significant interaction with time (Wald *χ*^2^ = 8.844, *p* = 0.012). *Post hoc* analysis revealed significantly greater improvement in forward-backward scores from T0 to T1 in the TCOG than in the CG (mean difference = 0.613, 95% CI: 0.156–1.070, *p* = 0.004). The difference between the TCG and the CG was not statistically significant (mean difference = 0.464, 95% CI: −0.040 to 0.969, *p* = 0.083).

The interaction between group and time for backward reach was significant (Wald *χ*^2^ = 10.368, *p* = 0.006). Post-hoc analysis indicated that the TCOG demonstrated significantly greater improvement in backward reach scores from T0 to T1 than the CG (mean difference = 0.921, 95% CI: 0.117–1.726, *p* = 0.018). The difference between the TCG and the CG was not statistically significant (mean difference = 0.857, 95% CI: −0.068 to 1.783, *p* = 0.080).

### 3.3 Secondary outcome measure results

Secondary outcomes were similarly evaluated at baseline (T0) and post-intervention. The main effects of group (Wald *χ*^2^ = 48.462, *p* < 0.001), time (Wald *χ*^2^ = 13.363, *p* < 0.001), and the group × time interaction (Wald *χ*^2^ = 52.241, *p* < 0.001) in the MoCA were all significant. Post-hoc analyses confirmed that MoCA score improvements from T0 to T1 in both intervention groups significantly exceeded those in the CG (TCOG vs. CG: mean difference = 7.387, 95% CI: 5.031–9.743, *p* < 0.001; TCG vs. CG: mean difference = 7.000, 95% CI: 4.663–9.338, *p* < 0.001).

The main effects of group (Wald *χ*^2^ = 17.292, *p* < 0.001), time (Wald *χ*^2^ = 16.548, *p* < 0.001), and the group × time interaction (Wald *χ*^2^ = 8.958, *p* = 0.011) on MMSE scores were all significant. *Post hoc* analyses revealed that the intervention groups demonstrated significantly greater MMSE score improvements from T0 to T1 than the CG (TCOG vs. CG: mean difference = 3.422, 95% CI: 1.222–5.623, *p* < 0.001; TCG vs. CG: mean difference = 3.071, 95% CI: 0.889–5.254, *p =* 0.002).

The main effect of group on the CSIT was significant (Wald *χ*^2^ = 27.077, *p* < 0.001), as was the group × time interaction (Wald *χ*^2^ = 17.866, *p* < 0.001). Post-hoc analyses revealed that both intervention groups demonstrated significantly greater improvements in CSIT scores from T0 to T1 than the CG (TCOG vs. CG: mean difference = 3.860, 95% CI: 2.280–5.440, *p* < 0.001; TCG vs. CG: mean difference = 3.286, 95% CI: 1.558–5.013, *p* < 0.001).

A significant interaction between group and time was observed for the GDS (Wald *χ*^2^ = 21.505, *p* < 0.001). *Post hoc* analysis indicated a significantly greater reduction in depressive symptoms from T0 to T1 in the TCOG than in the CG (mean difference = −1.461, 95% CI: −2.776 to −0.145, *p =* 0.024).

The group × time interaction for the PSQI was significant (Wald *χ*^2^ = 8.095, *p* = 0.017). *Post hoc* comparisons revealed that the TCOG exhibited a significantly greater reduction in PSQI scores (indicating improved sleep quality) from T0 to T1 compared with the CG (mean difference = −2.042, 95% CI: −3.891 to −0.193, *p* = 0.008) and the TCG (mean difference = −1.470, 95% CI: −2.984 to −0.043, *p* = 0.020). The difference between the TCG and the CG was not statistically significant (mean difference = −0.571, 95% CI: −2.636 to 1.493, *p* = 0.508).

### 3.4 Intervention safety and adverse events

During the 26-week intervention period, systematic safety monitoring revealed no cases of odor-induced discomfort, allergic responses, or withdrawal due to fatigue in the TCOG. Two TCOG participants reported mild, transient adverse effects (e.g., light-headedness) during early sessions, with symptoms resolving spontaneously without intervention modification. Comprehensive safety assessment confirmed no other intervention-related adverse events across all study groups.

## 4 Discussion

This study investigated the effects of combined eight-form Tai Chi and olfactory stimulation on working memory in older adults with MCI. Tai Chi training alone and the combined intervention of Tai Chi with olfactory stimulation effectively improved working memory, overall cognitive function, and sleep quality in this population. Notably, the TCOG demonstrated significantly greater gains than the TCG in digit span tasks and sleep quality. This result suggests that the combined intervention may yield broader beneficial effects across multiple cognitive and behavioral dimensions than a single intervention. The combined intervention appears to selectively enhance working memory and executive function, cognitive domains supported by the prefrontal-hippocampal circuitry. This finding is reinforced by observed improvements in sleep quality, which may promote memory consolidation and executive control.

The observed cognitive improvements in this study may be explained by synergistic mechanisms integrating Tai Chi with olfactory stimulation. As a multimodal mind–body intervention that incorporates moderate-intensity aerobic exercise, motor coordination, respiratory regulation, and focused attention training, Tai Chi may exert beneficial effects on working memory through synergistic pathways. Research suggests that Tai Chi can enhance synaptic function and neurogenesis by promoting neuroplasticity within the prefrontal–hippocampal circuit and increasing BDNF expression, thereby providing a neural foundation for working memory (17, 27). The emphasis on deep abdominal breathing and focused intention regulates autonomic nervous system balance, alleviates the detrimental effects of chronic stress on cognition, and optimizes cognitive readiness (5, 6, 28). Olfactory stimulation, as a non-invasive sensory intervention, directly activates the olfactory bulb–amygdala–hippocampus pathway, participating in emotional regulation and the encoding and storage of episodic memory (29, 30). Specific scents, such as rosemary and lavender, have been demonstrated to modulate brainwave activity and neurotransmitter release, thereby enhancing the efficacy of memory-related neural circuits. Note that although the aforementioned mechanisms are supported by research, their specific roles in the current intervention remain hypothetical as this study did not include neuroimaging or biomarker assessments. Future studies should employ multimodal assessment approaches to validate these proposed mechanisms.

Compared with previous studies, the innovation of this research lies in combining standardized eight-form Tai Chi with systematic olfactory stimulation to construct an integrated “exercise–perception–cognition” intervention model. This approach achieves multi-target, multi-pathway synergistic regulation at the mechanistic level. The design responds to current calls for multimodal integration strategies in MCI intervention research, addressing the limitations of single-modality studies in terms of overall efficacy and complementary mechanisms. The findings not only validate the feasibility of Tai Chi as an effective non-pharmacological intervention for MCI, consistent with reports by Liu et al. (31) and Wayne et al. (6), but likewise suggest the potential additional benefits from combined olfactory stimulation, offering novel therapeutic perspectives for MCI (28, 32).

However, this study has certain limitations. The sample origin was relatively homogeneous, and the sample size was limited, which may have affected the generalizability of the results. The absence of a neutral scent or placebo diffuser control also prevents definitive dissociation of the specific olfactory effects from broader placebo components, particularly for subjective secondary outcomes. Additionally, although the substantial between-group differences in cognitive measures suggest effects beyond test–retest learning, the lack of alternate test forms means practice effects cannot be entirely ruled out. These promising effect sizes should therefore be interpreted cautiously, and their clinical relevance confirmed in future studies utilizing parallel test versions. Given the specific nature of the intervention, a fully masked study was challenging to implement, which may have introduced bias because of expectations. This study primarily relied on behavioral indicators and did not include support from neuroimaging or biochemical markers, such as BDNF or cortisol, which leaves the explanation of the mechanism at an inferential stage (33). The intervention period was brief, and no long-term follow-up was conducted, precluding the assessment of sustained efficacy or the potential effects on MCI progression.

Future research may integrate functional magnetic resonance imaging and functional near-infrared spectroscopy to elucidate the neurological mechanisms involving combined interventions of brain networks. Regarding intervention strategies, the systematic exploration of optimal matching patterns between different olfactory stimulus parameters (e.g., type and concentration) and Tai Chi interventions could provide evidence for developing personalized protocols. Expanding the intervention scope to include multi-sensory combinations (visual and auditory) may test the universality and synergistic potential of such approaches. Large-scale, multicenter, long-term randomized controlled trials should also be conducted to validate the long-term efficacy and clinical translation value of this combined strategy.

In summary, the combination of eight-form Tai Chi with olfactory stimulation demonstrated positive effects in improving working memory, cognitive function, and sleep quality among older adults with MCI, with the combined intervention proving superior to Tai Chi training alone. This research provides novel insights into non-pharmacological interventions for MCI, although their mechanisms and efficacy require further validation and refinement through high-caliber studies.

## 5 Conclusion

Single intervention with eight-form Tai Chi and combined intervention with olfactory stimulation effectively enhanced working memory and overall cognitive function in older adults with MCI. The combined intervention demonstrated significant advantages in memory consolidation and alleviating depressive symptoms while also exhibiting additional benefits over standalone Tai Chi training in improving sleep quality. This integrated approach offers a promising new pathway for non-pharmacological intervention in MCI. However, its clinical application potential requires further validation through large-scale, long-term studies.

## Data Availability

The raw data supporting the conclusions of this article will be made available by the authors, without undue reservation.
