# Supplementary figures and images for: Effect of eight-form Tai Chi combined with olfactory stimulation on working memory in older adults with mild cognitive impairment
Source: Front Public Health. 2026 Jan 6;13:1724647. doi: 10.3389/fpubh.2025.1724647 (PMC12815704; doi:10.3389/fpubh.2025.1724647)

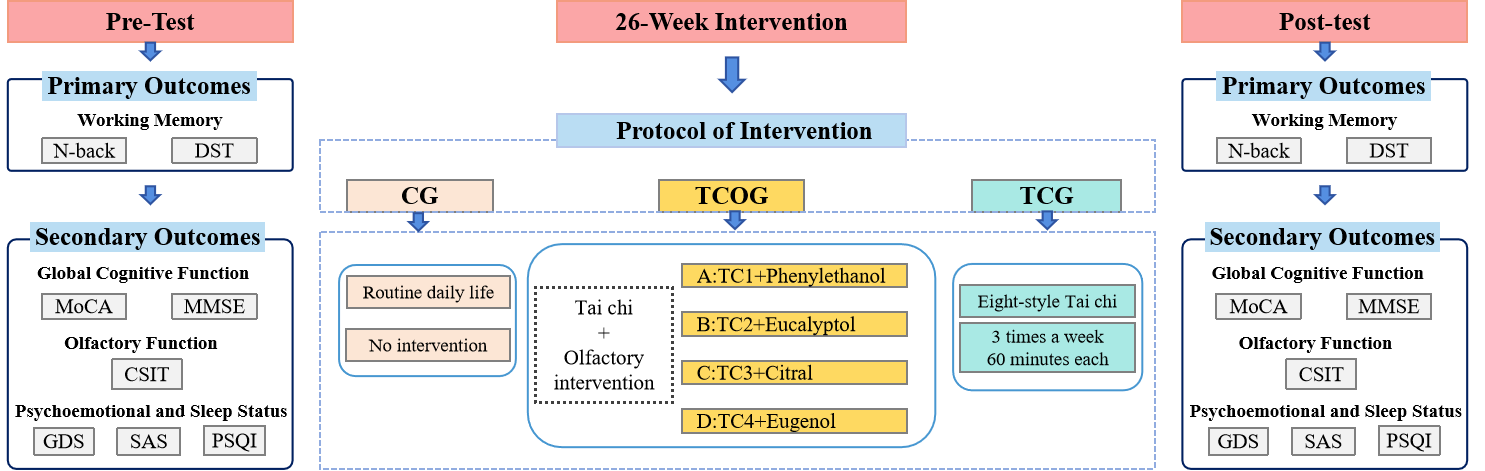

Supplement: Supplementary file 1 [file Image_1.PNG]
